# Inhaled tobramycin solution-associated recurrent eosinophilia and severe persistent bronchospasm in a patient with cystic fibrosis: a case report

**DOI:** 10.1186/1471-2431-7-11

**Published:** 2007-03-02

**Authors:** Roberto P Santos, Emad Awa, Ran D Anbar

**Affiliations:** 1 Department of Pediatrics, University Hospital, State University of New York Upstate Medical University, 750 East Adams Street, Syracuse, New York 13210, USA

## Abstract

**Background:**

Delivery of tobramycin by inhalation to the lungs of patients with cystic fibrosis (CF) who are infected with *Pseudomonas aeruginosa *has been proven to be effective and safe. The aerosol administration allows high concentrations of tobramycin to be delivered to the site of infection with limited systemic absorption. In rare patients, systemic absorption of inhaled tobramycin may be significant enough to produce toxic effects, such as renal and vestibular toxicities.

**Case presentation:**

We report a patient with CF who developed recurrent eosinophilia and severe persistent bronchospasm following repeated administration of preservative-free tobramycin by inhalation, beginning at 16 months of age. Also, he developed similar signs and symptoms when he was administered tobramycin intravenously on one occasion at 5 1/2 years. The patient had a history of environmental allergies. Temporal sequence of his signs and symptoms after each administration of tobramycin (similar to re-challenge testing), and his improvement after discontinuation of the drug strongly suggest an adverse drug reaction.

**Conclusion:**

Hypersensitivity reaction should be considered in patients who develop recurrent eosinophilia and deterioration of pulmonary function following the use of tobramycin by inhalation or by intravenous administration.

## Background

The delivery of tobramycin by inhalation to the lungs of patients with cystic fibrosis (CF) who are infected with *Pseudomonas aeruginosa *has been proven to be effective and safe [1]. The aerosol administration allows high concentrations of tobramycin to be delivered to the site of infection with limited systemic absorption. The portion of inhaled tobramycin available for systemic absorption includes approximately 10% of the total dose that is delivered to the lungs, and some that gets to the oropharynx and eventually swallowed [1].

There is poor systemic absorption of aerosolized tobramycin into the lungs among patients with CF, and its absorption from alveolar space into the pulmonary circulation is rate-limited [2]. Several studies have consistently documented low serum concentrations of tobramycin following its aerosolized administration [1]. Rarely, renal [3] and vestibular toxicities [4] have been described following inhaled tobramycin. A single case report describes development of a rash following inhaled tobramycin, in a patient who had developed a similar rash following a course of intravenous gentamicin and piperacillin-tazobactam [5]. These examples illustrate that systemic absorption of inhaled tobramycin may be significant enough to produce toxic effects among patients with CF. Inhaled tobramycin has been associated with cough and alteration of voice [6,7]. Development of acute bronchospasm has been associated with use of intravenous preparations of tobramycin for aerosol administration. This effect is thought to be related to antioxidants and preservatives contained within these preparations [1]. Thus, bronchospasm is less likely to occur with use of TOBI^® ^[6,7], the preservative free tobramycin solution for inhalation (TSI) that has been licensed by the United States Food and Drug Administration.

Eosinophilia has been reported to be associated with intravenous administration of tobramycin, including a patient treated for Gram-negative bacteremia [8], and among nine of 88 patients who received tobramycin in combination with cefamandole for peritonitis [9]. Furthermore, a patient with end-stage renal disease who was treated for sepsis with parenteral tobramycin and vancomycin developed persistent fever, eosinophilia and rash. Although temporal sequence had suggested vancomycin as the probable cause [10], the possible contribution of tobramycin could not be totally excluded.

We report a patient with CF who developed recurrent eosinophilia and severe persistent bronchospasm following repeated administration of TOBI^®^.

## Case Presentation

The patient was diagnosed as having CF, based on prenatal testing that was performed because he had a brother with CF. His CF transmembrane conductance regulator (CFTR) protein genetic analysis showed homozygosity for the ΔF508 mutation. At a year of age, his treatments included pancreatic enzymes for his pancreatic insufficiency; a multivitamin; nebulized albuterol and cromolyn sodium in the treatment of occasional coughing and wheezing that were thought to be attributable to hyperreactive airways; and daily dornase alfa, which has been used routinely as preventive therapy in infants at our Cystic Fibrosis Center since 1996.

At 16 months of age, the patient presented with wheezing, and was found to harbor *Pseudomonas aeruginosa *in his deep throat culture for the first time. He was prescribed a one-month course of preservative-free TSI, 150 mg/2.5 mL twice daily by nebulization. With this therapy, his cough and wheezing improved, only to worsen when the TSI was discontinued. Therefore, he was started on every other month administration of TSI that appeared to be recurrently associated with improvement in his respiratory status, even though *Pseudomonas *was not again documented in his throat cultures at that time. A year later his TSI dose was increased to 300 mg/5 mL twice daily.

At 2 years of age, as part of a routine screen, the patient was noted to have an elevated serum IgE level of 843 IU/mL (normal range, 2–35 IU/mL), and his eosinophil count (11%) was increased several fold (Table 1). The possibility of allergic bronchopulmonary aspergillosis was considered. However, anti-*Aspergillus *antibodies (< 1:8 by complement fixation) as well as skin scratch testing for *Aspergillus *were negative. As the patient's eosinophilia persisted (15–20%), further investigations over the subsequent three years included a re-check of *Aspergillus *antibody titers (including *A. fumigatus, A. flavus, and A. niger*), stool sample for ova and parasites (including wet mount, permanent stain and modified acid fast stain), *Giardia *antigen in stool, and *Toxocara canis *antibody levels; all were negative. His radioallergosorbent test (RAST) for common allergens in the northeastern part of the United States showed low level response to *Alternaria alternata *(77%) and to *D. farinae *(71%). A chest radiograph was clear.

The patient continued to suffer from recurrent wheezing and therefore was prescribed nebulized budesonide, oral montelukast, and infrequent 3–5 day bursts of oral prednisolone. When the patient was nearly 5 years old he began performing pulmonary function testing (PFT) on a routine basis, and demonstrated normal lung function, without improvement following bronchodilator administration, during a month while he was off TSI (Table 2). As he had not been found to harbor *Pseudomonas *for more than three years, it was decided to stop his every other month TSI.

At 5 1/2 years the patient developed purulent nasal drainage, coughing and wheezing. At that time, his deep throat culture revealed *Staphylococcus aureus *and *Serratia marcescens*. As he did not respond to therapy with oral amoxicillin/clavulanate, ciprofloxacin, azithromycin, and prednisone, he was hospitalized and treated with intravenous ceftazidime and tobramycin. His symptoms improved over the first few days of the hospitalization, but then he developed increased wheezing and dyspnea. His pulmonary function testing revealed a severe obstructive pattern, which improved with bronchodilator administration (from a forced expiratory volume in 1 second of 27% to 34% of predicted based on his height and age). He also had eosinophilia (13%) documented while he was receiving the intravenous antibiotics. A chest radiograph revealed a slight right middle lobe infiltrate. He was treated with a two week course of oral prednisone, with rapid improvement in his symptoms. His pulmonary function improved within a week and his eosinophils normalized about a week off intravenous tobramycin. His chest radiograph then revealed clear lung fields.

In further work-up of the patient's recurrent eosinophilia, the possibility of a fungal sinus infection was proposed by an Infectious Disease consultant. Therefore, the patient underwent computed tomography studies of his sinuses that revealed bilateral opacification of the maxillary sinuses. A sinus biopsy was consistent with chronic sinusitis. The sinus specimen cultures revealed *Alcaligenes xylosoxidans*, but were negative for fungus and acid fast organisms. Because of the apparent worsening of his chronic clinical condition half a year after discontinuation of every other month TSI therapy, and given the presence of the Gram-negative organisms *Serratia *and *Alcaligenes *in his airways, the patient was restarted on every other month TSI.

He developed recurrent wheezing once his prednisone was discontinued. He was subsequently started on every other day prednisone, at a dose of 0.8 mg/kg/day, which helped control his wheezing. At 6 years TSI was discontinued as he was found to harbor *Alcaligenes xylosoxidans *in his deep throat culture, which was resistant to tobramycin.

He did well until 6 1/2 years when he developed an upper respiratory infection with associated sinusitis and coughing. A throat culture demonstrated *Pseudomonas aeruginosa *for the first time since his early childhood for which he was prescribed oral ciprofloxacin and TSI. Within several days, he developed severe wheezing with an associated severe obstructive pattern on pulmonary function testing with eosinophilia (14%). At that time, review of his medical record revealed a strong relationship between use of tobramycin and the patient's eosinophilia and bronchospasm (Tables 1 and 2). This prompted permanent discontinuation of the patient's TSI.

Over the subsequent 12 months, the patient did not demonstrate eosinophilia. He tolerated weaning off chronic prednisone by the age of 7 years. He continued to have elevated serum IgE levels, however was noted to be trending down while off TSI (Table 2). He has had recurrent mild bronchospasm, although he has not had further episodes of severe airway obstruction.

## Discussion

Tobramycin associated recurrent eosinophilia with severe persistent bronchospasm has not been reported previously. The temporal sequence of the patient's signs and symptoms, after every administration of TSI or tobramycin intravenously, strongly suggest an adverse drug reaction (ADR).

An ADR is a noxious and unintended response to a drug at therapeutic doses [11] – in our patient, documented as eosinophilia and severe obstructive airway disease after every administration of tobramycin. The Naranjo scale confirms tobramycin as the highly probable cause of his ADR. This is a reliable (inter-raters [r = 0.92] and intra-raters [r = 0.91] reliability, p < 0.001) and valid (with consensual, content and concurrent validity) probability scale consisting of a simple questionnaire used to assess ADR [12]. Although his serum IgE remained elevated, there was no recurrence of eosinophilia after discontinuation of the TSI. He has had episodes of bronchospasm; however, not as severe as when he was receiving TSI. Furthermore, other emergent causes of his signs and symptoms (such as allergic bronchopulmonary aspergillosis and parasitemia) have been excluded.

Hypersensitivity reaction to tobramycin is a plausible explanation in our patient. This has been reported in an adult patient with CF who developed urticaria, pruritis, and difficulty of breathing after the third dose of intravenous tobramycin [13]. In our patient, hypersensitivity reaction is supported by his history of environmental allergies.

The patient might have benefited from a diagnostic bronchoscopy when he developed unexplained respiratory symptoms with associated eosinophilia. This procedure might have documented eosinophilia, *Pseudomonas*, fungus, or another infectious organism in the bronchoalveolar lavage fluid, and such findings could have guided his therapy.

There are *in vitro *data to suggest that aminoglycoside accumulation in non-CF cells is under regulation by effectors and inhibitors of the CF transmembrane conductance (CFTR) channel. This regulation is lost among CF cells with ΔF508 mutations with inhibition of exocytosis since the CFTR channel is inoperable. This results in accumulation of aminoglycoside within the affected cells [14]. As patients exposed to higher drug concentrations are more at risk for ADR, it is possible that our patient had significant tissue accumulation of tobramycin leading to his symptoms.

Any drug has the potential to produce an ADR. Medical personnel taking care of children with complicated medical conditions such as CF, need to maintain a high index of suspicion in differentiating complications of the primary illness from an unexpected consequence of prescribed medications [15].

## Conclusion

Hypersensitivity reaction should be considered in patients who develop recurrent eosinophilia and deterioration of pulmonary function following the use of tobramycin by inhalation or by intravenous administration.

## Competing interests

The author(s) declare that they have no competing interests.

## Authors' contributions

RS, EA, and RA wrote the case report. RA treated the reported patient, and edited the report. All authors read and approved the final manuscript.

## Pre-publication history

The pre-publication history for this paper can be accessed here:



## Figures and Tables

**Table 1:** Summary of TSI administration, pulmonary status, and hematologic profiles before 5 years

	**Before TSI Therapy**	**TSI started 150 mg/2.5 mL twice daily**	**On TSI 150 mg/2.5 mL twice daily**	**TSI Increased to 300 mg/5 mL twice daily**	**On TSI 300 mg/5 mL twice daily**
**Age**	6 months	16 months	2 years	2 1/2 years	4 years
**Pulmonary Status**	No wheezing	(+) wheezing; 1^st ^(+) throat culture for *P. aeruginosa*	(+) wheezing	(+) wheezing	(+) wheezing
**WBC (/mm**^3^**)**	15.2	NA	9.1	12	11.2
**Eosinophils (%)**	1	NA	11	15	20
**IgE (IU/mL)**	NA	NA	843	NA	299

Abbreviations: TSI, tobramycin solution for inhalation; WBC, white blood cell count; NA, not available

**Table 2:** Summary of TSI administration, pulmonary function, and hematologic profiles at 5 years and after

	**Off TSI**	**Intravenous tobramycin ~1 week**	**~1 weeks post intravenous tobramycin**	**Off TSI**	**On TSI 300 mg/5 mL twice daily**	**Off TSI**	**Off TSI**
**Age**	5 years	5 1/2 years	5 1/2 years	6 years	6 1/2 years	7 years	7 1/2 years
**Pulmonary Function**	FVC 105% FEV_1 _90%	FVC 45% FEV_1 _27%	FVC 76% FEV_1 _64%	FVC 103% FEV_1 _93%	FVC 49 % FEV_1 _32 %	FVC 90% FEV_1 _81%	FVC 91% FEV_1 _78%
**WBC (/mm**^3^**)**	7.9	16	15.8	NA	17.7	13.5	9.9
**Eosinophils (%)**	2	13	1	NA	14	0	1
**IgE (IU/mL)**	123	165	NA	NA	205	306	166

Abbreviations: TSI, tobramycin solution for inhalation; WBC, white blood cell count; FVC, forced vital capacity; FEV_1_, forced expiratory volume in 1 second; NA, not available
